# Enhanced Dye‐Sensitized Mechanosensation Utilizing Pulsed and Digitally Modulated Light

**DOI:** 10.1002/advs.202403690

**Published:** 2024-08-19

**Authors:** Tarek Rafeedi, Laura L. Becerra, Nicholas Root, Yi Qie, William Brown, Baiyan Qi, Lei Fu, Guillermo Esparza, Lekshmi Sasi, Kabir Kapadia, Romke Rouw, Jesse Jokerst, Darren J. Lipomi

**Affiliations:** ^1^ Department of Nanoengineering University of California San Diego 9500 Gilman Drive La Jolla CA 92093 USA; ^2^ Department of Psychology University of Amsterdam Nieuwe Achtergracht 129‐B WT Amsterdam 1018 Netherlands

**Keywords:** dye sensitizer, haptic perception, incoherent light, photoacoustics, tactile

## Abstract

The generation of pressure perturbations in matter stimulated by pulsed light is a method widely recognized as the photoacoustic or light‐induced thermoelastic effect. In a series of psychophysical experiments, the robustness of the tactile perception generated with a variety of light sources is examined: a diverging pulsed laser used for photoacoustic tomography optical parameter oscillation (OPO), a miniature diode laser (MDL), and a commercial digital light processing (DLP) projector. It is demonstrated that participants can accurately detect, categorically describe the sensations, and discern the direction of pulsed light travel. High detection accuracy is reported as follows: (*d*′ = 4.95 (OPO); *d*′ = 2.78 (modulated MDL); *d*′ = 2.99 (DLP)) of the stimulus on glabrous skin coated with a thin layer of dye absorber. For all light sources, the predominant sensation is felt as vibration at the distal phalanx (i.e., fingertip, 55.21–57.29%) and the proximal phalanx (41.67–44.79%). At the fingertip, thermal sensations are perceived less frequently than mechanical ones. Moreover, these haptic effects are preserved under a wide range of pulse widths, spot sizes, optical energies, and wavelengths of the light sources. This form of sensory stimulation demonstrates a generalizable non‐contact, non‐optogenetic, in situ activation of the mechanosensory system.

## Graphical abstract

Humans primarily perceive vibration sensations from pulsed light. A thin layer of dye, opaque or translucent, on the skin enhances the tactile effect. The produced robust effect is achieved with visible and invisible light wavelengths, and with incoherent light from a commercial digital projector.

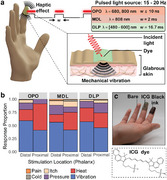

## 1 Introduction

Most experimental and commercial systems for stimulating the sense of touch require physical contact with an actuator embedded in, e.g., a smartphone, video game controller, or wearable device. However, there are many contexts—e.g., the laboratory, clinic, defense technologies, and consumer electronics—where it may be desirable to activate peripheral neurons remotely; that is, to “project” haptic signals onto the skin. Here, we used pulsed light from a range of sources, notably a conventional digital projector controlled using a laptop, to generate “photoacoustic” signals in the skin. In psychophysical experiments, a cohort of human participants perceived these optical signals as predominantly mechanical—as opposed to thermal or noxious. While a small number of research articles have demonstrated a similar effect using short (*ns* − µ*s*), powerful bursts of focused coherent light from a laser,^[^ ^1^, ^2^ ^]^ this is the first time the effect has been demonstrated with desktop, easily available equipment.

### 1.1 The Photoacoustic Effect

The photoacoustic effect, discovered in the 1880s, involves the generation of pressure waves in matter using pulsed light.^[^ ^3^ ^]^ The effect has been used for decades in materials research to measure dissipation and storage mechanisms in solids,^[^ ^4^, ^5^, ^6^ ^]^ and more recently to generate high‐frequency ultrasound to image and map tissue using endogenous absorbers, e.g., hemoglobin, or exogenous absorbers.^[^ ^7^, ^8^, ^9^ ^]^ The penetration depth of red or NIR light, along with the lack of scattering experienced by acoustic waves in biological tissue, has made photoacoustic tomography a robust tool for imaging with high resolution at depths of centimeters.^[^ ^10^ ^]^

Photoacoustic waves are initiated by a thermal expansion pulse, caused by the absorption of optical energy and photothermal conversion. The expansion pulse decays into a wide range of frequencies which depend in part on the width of the optical pulse (nanoseconds in biomedical imaging).^[^ ^11^, ^12^, ^13^, ^14^, ^15^ ^]^ In the context of biomedical imaging, the duty cycle is small enough—often substantially less than 1% —that fast dissipation of thermal energy limits accumulation of heat, and thus induces rapid thermal expansion and subsequent contraction.^[^ ^16^ ^]^ The stress waves created by this event contain a range of frequencies in the tens of kHz to MHz regime, too high to be detected by afferents of the skin. However, the individual light pulses generating these waves can be repeated at rates that are well within the frequency range of detection of mechanoreceptors, i.e., 20 Hz to 1 kHz.^[^ ^17^ ^]^

The physical phenomena governing thermoelastic pulses and photoacoustic waves in matter have been investigated in detail. In particular, modeling and simulation studies of this effect in tissue and skin have been examined extensively from the standpoint of biomedical imaging.^[^ ^18^, ^19^, ^20^ ^]^ Generating pressure waves in condensed matter depends on the optical and mechanical properties of the target, as well as the properties of the incident light. The pressure change at position **r** in such instances is given by

(1) $$p_{0}\left(r\right)=\frac{B\beta \mu_{a}\left(r\right)\psi \left(r\right)}{\rho c_{p}}$$

where *B* is the bulk modulus of the target, β is the thermal expansion coefficient, μ_*a*_ is the absorption coefficient, ψ is the pulse fluence, ρ and *c*_*p*_ are the density and the constant‐pressure specific heat capacity of the target, respectively.^[^ ^21^ ^]^ Increasing the absorption coefficient for the target by the application of dye effectively increases the magnitude of the heat conversion of light which leads to a higher initial pressure generation. However, the aforementioned pressure description relies on the conditions of thermal confinement and acoustic stress confinement, described by the time constants:

(2) $$\tau_{a}=\frac{L}{c}$$

(3) $$\tau_{th}=\frac{L^{2}}{\alpha }$$

where *L* is the characteristic length of the illuminated target, *c* is the acoustic velocity in the medium, and α is the thermal diffusivity of the target medium. Achieving high‐resolution imaging in photoacoustic tomography requires a pulse width that is smaller than the stress Equation (2) and thermal confinement Equation (3) time constants. Such pulse parameters (on the order of nanoseconds for human tissue) can be achieved using an optical parametric oscillator (OPO). However, Gao et al. demonstrated that creating a pressure pulse in a target is possible using pulse widths higher than the confinement parameters^[^ ^22^ ^]^ and describe the dependence of pressure peaks on pulse width and confinement parameters in such cases.

The subsurface acoustic waves have been measured in stained hydrogels and elastin biomaterials brushed with black ink^[^ ^23^ ^]^ or with indocyanine green dye (ICG).^[^ ^24^ ^]^ The pressure signals generated at various depths were detected by a transducer placed at the opposite end of the sample ≈1 mm in thickness. The highest peak pressures were generated at the material surface with a gradual decline, due to Beer's law or the dye diffusion profile. Given these observations, the photoacoustic effect on inked human skin is likely to produce a similar pressure profile along the beam direction.

### 1.2 Tactile Perception and Articulation

Mechanical forces exerted at the surface of the skin are translated to neural impulses by the cutaneous end organs and afferent fibers of the somatosensory system.^[^ ^25^, ^26^ ^]^ Along this pathway, signals are integrated, encoded, and ultimately categorized as familiar conscious percepts, e.g., pressure, vibration, stretch, and pain. In natural environments, the mechanical force applied to the surface of the skin must cause sufficient deformation to initiate action potentials stemming from the end organ or free nerve ending. Low‐threshold mechanoreceptor axons innervate their respective end organs and conduct signals that pertain to their receptor type and the magnitude and frequency of the deformation. The mechanosensory neurons of the human skin can in principle detect mechanical signals with frequencies below approximately 1500 Hz. For example, the Pacinian corpuscle, primarily responsible for sensing vibrations on the skin, is most sensitive around 250 Hz.^[^ ^25^ ^]^ The Meissner's corpuscle, responsible in part for sensations of fine touch, is most sensitive in the tens of Hz, and the Merkel cell‐neurite complex is most sensitive at the level of a few Hz.^[^ ^27^, ^28^ ^]^

Studies have explored various methods for delivering cutaneous tactile information without direct contact.^[^ ^29^ ^]^ Primary approaches have included exerting mid‐air pressure against the skin by air streams and air vortex rings. For example, Gurocak et al. employed air jets to create thrust forces on the hands of subjects to produce the sensation of weights of objects in virtual reality.^[^ ^30^ ^]^ Gupta et al. generated and tuned the formation of toroidal air vortices to direct tactile stimuli to different regions of the human body.^[^ ^31^ ^]^ While these pressure generation methods have successfully delivered haptic feedback, they are limited by spatial and temporal resolution, as well as the distance over which the signals can be transmitted. Alternatively, ultrasound haptic devices have been extensively investigated in their ability to evoke tactile sensations in mid‐air. Focused ultrasound waves generate acoustic radiation pressure, enabling the transfer of haptic cues. This ultrasound‐based approach facilitates applications such as haptic textures,^[^ ^32^ ^]^ localized haptic feedback,^[^ ^33^ ^]^ and haptic holography.^[^ ^34^ ^]^

**Figure 1:**
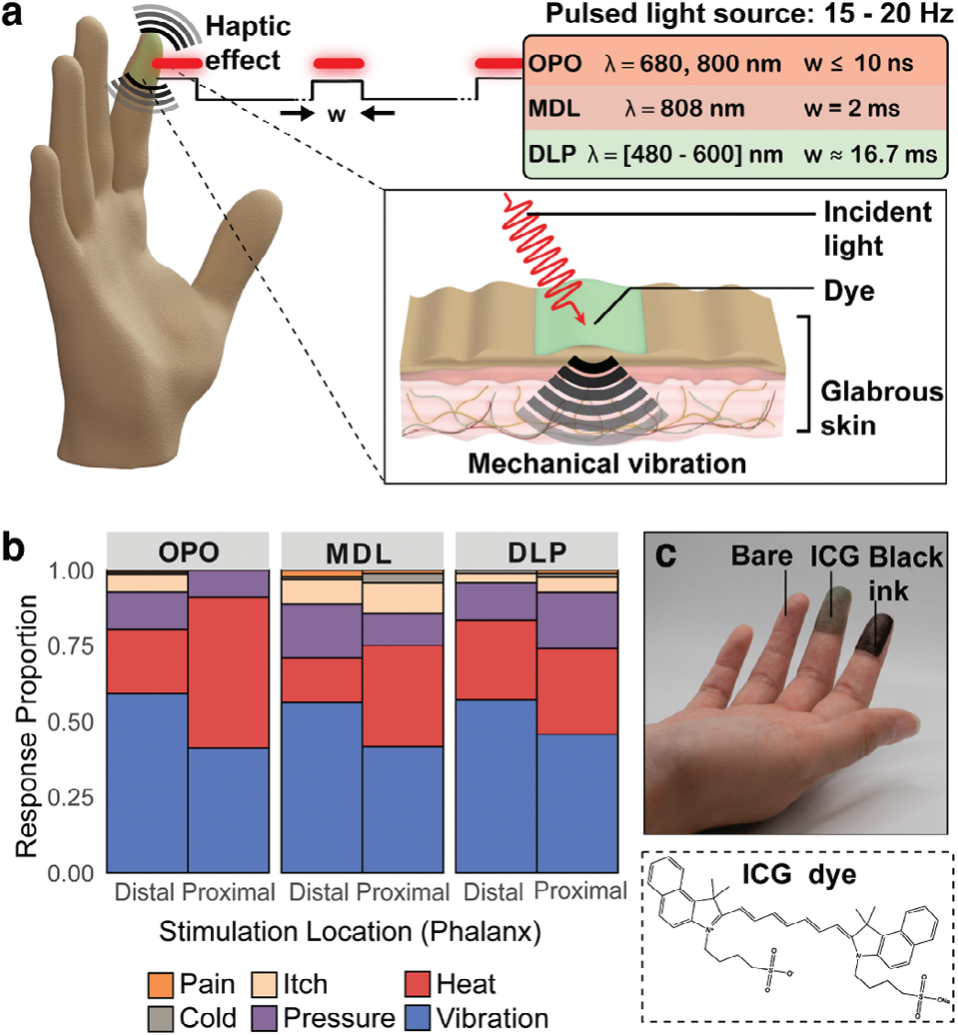
A brief overview of the effect and key result described in this work. a) Schematic diagram of the use of pulsed light to generate tactile sensations mediated by the photoacoustic effect. Light incident on the surface generates a mechanical effect detected by cutaneous receptors. b) Summarized proportions of tactile sensations reported by human participants after applying the optical stimulus on the distal (fingertip) and the proximal phalanges using various light sources. c) Photograph of a participant's fingertips coated with dyes used to sensitize the skin to the photoacoustic effect. The bottom panel shows the chemical structure of the IR‐absorbing dye, indocyanine green (ICG).

Lasers have recently emerged as candidates for generating cutaneous sensations. Historically, lasers were found to induce nociception and pain when applied directly to body surfaces.^[^ ^35^, ^36^ ^]^ When mediated through air, laser light can create plasma and acoustic fields in mid‐air and produce tactile sensations on human skin via shock waves.^[^ ^37^, ^38^ ^]^ In the last decade, it has been demonstrated that pulsed single‐wavelength lasers can evoke tactile sensations on the skin mediated by thermoelastic effects. Incident pulsed light is converted into thermal energy, inducing thermal expansion and subsequent relaxation. This process generates stress (acoustic) waves either directly on the skin or through an elastic medium (i.e., the photoacoustic effect^[^ ^1^, ^2^ ^]^). While laser‐based stimuli can pose a safety risk to the eyes, this issue can be overcome by combining the stimuli with virtual reality headsets that cover the eyes. Additional investigation can be done to explore the possibility of using constructive interference of lower power light sources. Despite the general understanding of the phenomena surrounding the photoinduced thermoelastic expansion of condensed matter, there has not been significant exploration as to the frequency and degree of irradiation and absorption that is necessary to drive perception in human subjects. Previous studies have shown that a single laser pulse, without the presence of a dye layer, can be detected as a mechanical stimulus by participants, described mostly as a “light touch” or “prickle.”^[^ ^1^ ^]^ Their simulation results on this effect suggested these thermoelastic deformations (both radial and axial) to be at about a few hundred nanometers, at depths of 100–200 µm within the skin. Apart from that, there is little experimentation done to explore the expected deformations and the cutaneous receptors responsible for their detection. The mechanical perceptibility of this effect is emphasized in previous work done by Lee et al. where a thermally insulating film was used to isolate the mechanical sensation.^[^ ^2^ ^]^ The effect was still detectable when presumably no light was absorbed by the skin itself. Here, we examine the effect of various light‐sources and optical absorbers (applied on the skin) in producing a robust tactile sensation, as shown in (**Figure** **1**a,b). We evaluate the usability of several light source systems with different power, pulse, and wavelength specifications, including incoherent light from an inexpensive DLP projector. We perform psychophysical studies to examine the effects of applied dyes and the robustness of the mechanical sensation, and to demonstrate the spatial localization or movement of the sensation.

## 2 Results

### 2.1 Psychophysical Experimental Design

We designed a sequence of psychophysical experiments to characterize the quality of perception (vibration vs heat vs pressure), different dye absorbers (Figure 1c), spatial effects (direction of movement of the stimulus), and light sources of different powers and wavelengths.^[^ ^39^ ^]^ In contrast to the reports by Jun et al.,^[^ ^1^ ^]^ pilot experiments with our system suggested that participants were unable to feel any effect from the pulsed light without the use of a dye. This may be due to the difference in divergence and absorptance of the particular wavelengths. However, when applied to the fingers, a thin film of black ink from a marker or translucent ICG dye allowed the perception of a strong effect in most participants.

The safety concerns of light sources—particularly lasers—are related to potential damage to the retina, burning of the skin, and the generation of reactive oxygen species. The OPO and MDL laser sources had divergent beams and the exposure was below the safety threshold according to ANSI limits for skin exposure when the distance from the source to the skin surface was 1.5 cm. We verified safety of skin at 1.5 cm distance for the OPO laser by calculating a fluence of 13 mJ cm^−2^, given the measured spot size of 2.7 cm^2^ and technical specification of 45 mJ for peak energy. The safety of the MDL mechanically chopped and modulated was verified at 1.5 cm away with a calculated fluence of 3.56 and 3.28 mJ cm^−2^, respectively. Note that in the mechanically chopped case, the fluence was comparable since the distance was 3 cm during examination, but had a larger pulse width. Lastly, the DLP projector fluence was estimated to be 10.3 mJ cm^−2^.

#### 2.1.1 Nanosecond Pulse OPO Laser Experiments

**Figure 2:**
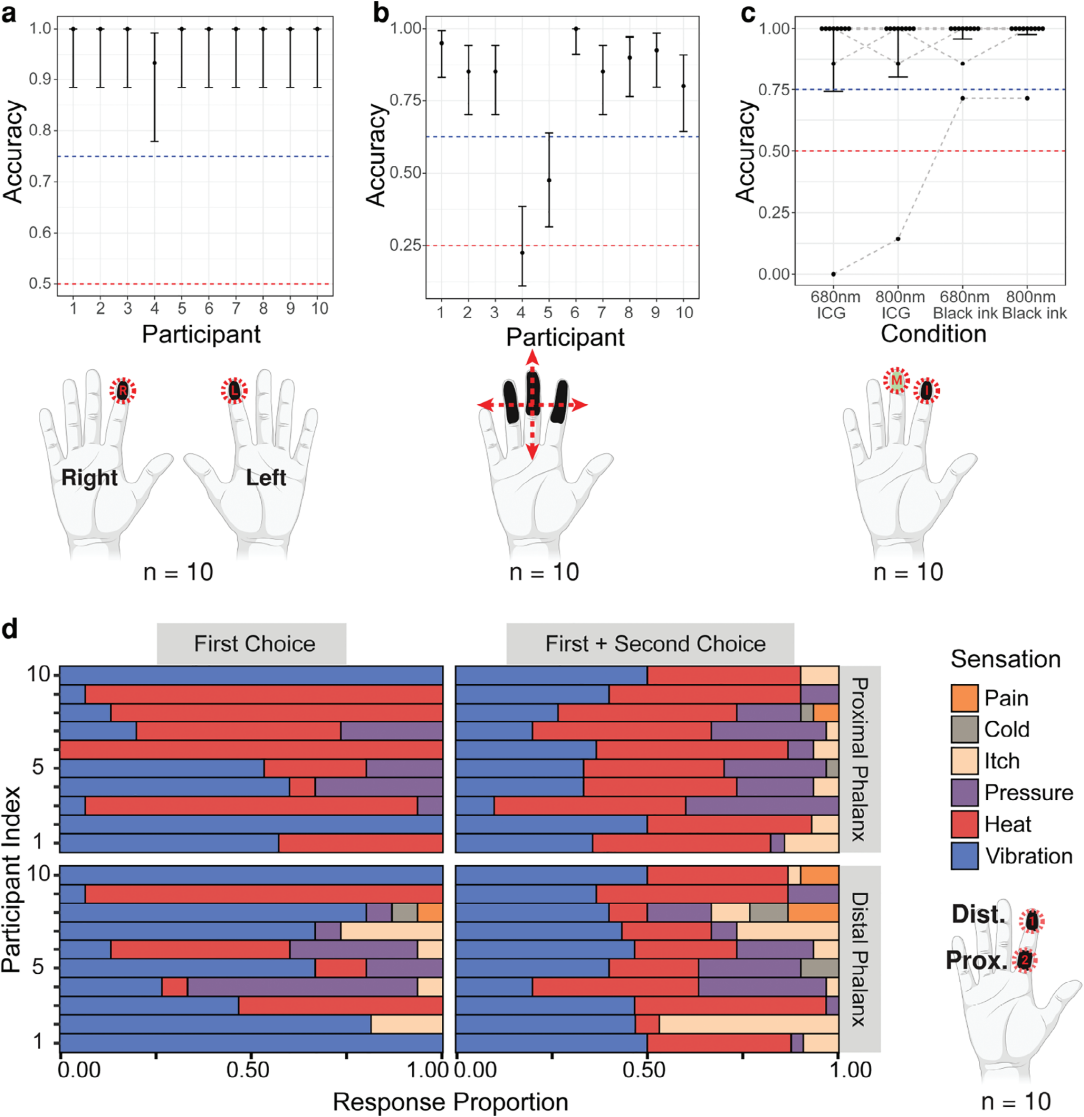
Perception accuracy of the effect in various conditions and effect categorization results produced using OPO system. a) Accuracy results of 2‐AFC experiment to test for detection of stimulus on the index fingers marked with black ink. b) Accuracy of 4‐AFC test for stimulus sweep in four different directions (red arrows) across distal and middle phalanges with black ink. c) Accuracy results for detection of stimulus with different wavelengths and ink types (GLMM estimated marginal means) (Table S2, Supporting Information). d) Proportion of responses for primarily perceived sensations by participants on the proximal and distal phalanges (left panels). Right panels show combination of primary and secondary perceived sensations for proximal and distal phalanges (top and bottom, respectively). Note: All error bars are 95% confidence intervals, which were back‐transformed from the logit scale. The red dotted lines indicate chance performance, and the blue dotted lines depicts the 75% accuracy level typically used in 2‐AFC (or 62.5% for 4‐AFC) experiments to define the detection threshold.

For the first battery of experiments (**Figure** **2**) the light source used was a nanosecond optical parametric oscillator (OPO) laser that is commonly used for photoacoustic imaging (Vevo F2 LAZR‐X). The first experiment was a two‐alternative forced choice (2‐AFC) test in which black ink was applied to the index fingertips of both hands (Exp A) (Figure S2a, Supporting Information). On each trial, the marked regions of one of the two fingertips were irradiated with 680 nm wavelength stimulus, to test if participants could identify which finger (left or right hand) was stimulated. Overall, participants performed significantly better than chance (χ^2^ (1) = 391.81, *p* < 0.001; *d*′ = 4.95, *d*′ 95% CI [3.94,5.96]; more details on statistical analysis in the Experimental Section). We also characterized individual participant performance; of the ten participants, nine were 100% accurate, and the tenth was accurate on 93% of trials (Figure 2a). This performance is significantly higher than the 50% that would be expected by chance (all *p* < 0.001), and is also higher than the 75% accuracy that is usually set by convention to correspond to the detection threshold in 2‐AFC experiments.

Next, we tested the ability of participants to discern the direction of travel of the pulsed beam on the skin. On each trial, we swept the pulsed beam over an inked portion of the dominant hand in one of four directions, and prompted the participants to identify the direction (Exp B) (Figure S2b, Supporting Information). Overall, participants performed significantly better than chance (χ^2^ (1) = 14.81, *p* < 0.001; *d*′ = 2.14, *d*′ 95% CI [1.42,2.80]). We also characterized individual performance of each participant: of the ten participants, one was not significantly more accurate than chance (*p* = 0.86), one was significantly more accurate than chance (*p* = 0.03) but not more accurate than the typical detection threshold (62.5% for 4‐AFC), and eight were significantly more accurate than chance (all *p* < 0.001) and also more accurate than the typical detection threshold (Figure 2b). The results from this experiment showed that participants could discern the direction of the sweep (i.e., left to right, front to back, etc.), suggesting that the relative position of the laser the stimulus can be spatially and temporally resolved.^[^ ^40^, ^41^ ^]^ Additionally, the experiment demonstrated the ability of participants to detect stimulation across multiple areas of the finger (distal and middle phalanges) and across multiple fingers.

We then sought to investigate whether the stimulus could be perceived with a near‐infrared (NIR) wavelength and a less conspicuous absorber than black ink. We thus turned to ICG, commonly used for tissue staining in biomedical imaging,^[^ ^42^, ^43^ ^]^ and compared the effect of both absorbers at both 680 and 800 nm (Exp B) (Figure S2c, Supporting Information). While overall accuracy was higher with black ink (Wald *Z* = 2.19, *p* = 0.03), this effect appears to have been driven entirely by the results of a single participant (Figure 2c). When the participant's data are excluded, performance was not significantly influenced by the absorber, wavelength, or their interaction (all *p* > 0.5). Crucially, in all four conditions, participants detected the stimulus more often than would be predicted by chance (all *p* < 0.05; see Tables S1 and S2, Supporting Information), and in all four conditions all but one participant surpassed the conventional detection threshold of 75% accuracy. Thus it appears that the generation of this “phototactile” effect is amenable to a variation in absorbers and wavelengths.

Pilot experiments and previous studies suggested that photoacoustic pulses could be perceived as a mechanical stimulus, rather than as, e.g., heat. However, the density of mechanoreceptors differs across different locations on the body.^[^ ^44^ ^]^ We conducted an experiment to determine which sensation was perceived and to also determine whether the type of sensation significantly differed by stimulation location (proximal and distal phalanges of the index finger). The relationship between stimulation location and reported sensation was highly significant (χ^2^(5) = 34.3, *p* < 0.001). When given a choice of pressure, vibration, itch, pain, heat, or cold, perceived by participants at the distal phalanx (fingertip), vibration was the most commonly reported sensation (Exp D) (Figure 2d; Figure S2d, Supporting Information). Post‐hoc analysis of the Bonferroni‐corrected χ^2^ residuals^[^ ^45^ ^]^ suggests that at the distal phalanx (vs proximal phalanx) there were significantly more reports of vibration (Z = 3.12, *p*_*adj*_ = 0.01) and itch (Z = 3.01, *p*_*adj*_ = 0.02), and significantly fewer reports of heat (Z = –5.24, *p*_*adj*_ < 0.001). We also asked participants for a secondary sensation they felt during stimulation. Heat was the most commonly reported secondary sensation for both distal (42.5%) and proximal (40.25%) phalanges.

### 2.2 Miniature Diode Laser (MDL) Experiments

#### 2.2.1 Mechanically Chopped Light Sources

**Figure 3:**
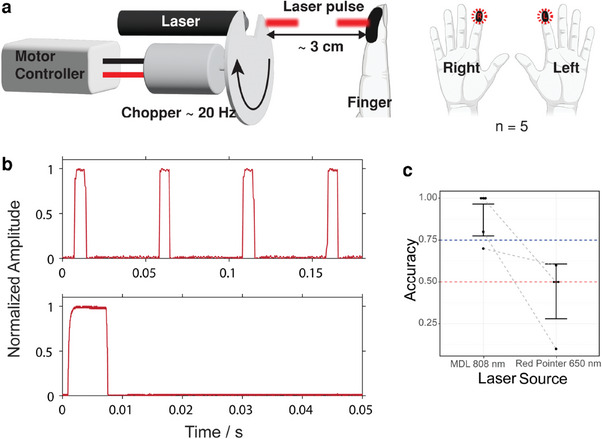
Perception accuracy of the effect using chopped low‐power lasers (MDL and red laser pointer). a) The custom handheld mechanical chopper rotated at 20 Hz to output b) periodic pulses. c) Discrimination accuracy results of 2‐AFC experiment of each source with inked fingers.

**Figure 4:**
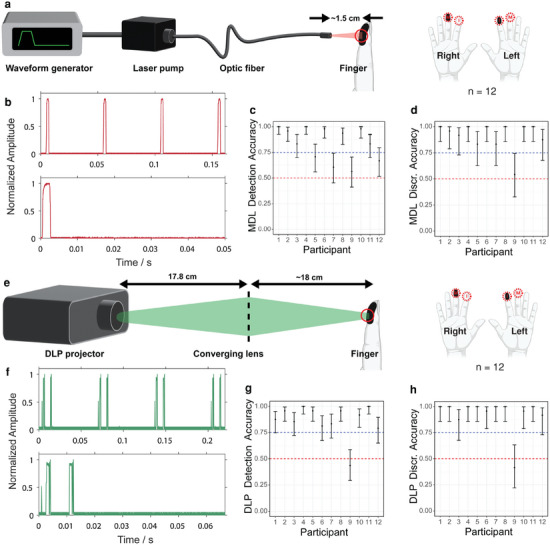
Schematics of the MDL laser and DLP projector systems, as well as the corresponding pulse characterization and observed perception accuracy. a) Schematic of the MDL set up. b) Normalized amplitude of the optical output of the modulated MDL lasers showing typical pulse train and single pulse profile. c) Accuracy results for the 2‐AFC detection of MDL stimuli. d) Accuracy results for the 2‐AFC discrimination of MDL stimuli. e) Schematic of the DLP projector stimulation apparatus. f) Normalized amplitude of the optical output of the modulated DLP showing the pulse train of dual pulses produced by the DLP system. g) Accuracy results for the 2‐AFC detection of DLP‐generated stimuli. h) Accuracy results for the 2‐AFC discrimination of DLP‐generated stimuli. Note: All error bars are 95% confidence intervals on estimated marginal mean of accuracy per condition, back‐transformed from the logit scale. The red dotted lines indicate chance performance, and the blue dotted lines depict the 75% accuracy level.

Given the expense (ca. $100 000) and inaccessibility of the medical OPO pulsed laser system, we investigated portable and less expensive light sources (**Figures** **3** and **4**). We pulsed two of the sources (a red laser pointer, 650 nm, and a more powerful miniature diode laser, MDL, 808 nm) at 20 Hz with 6.25 ms pulse width. Using a handheld setup (Figure 3; Figure S6, Supporting Information), we first conducted a simple 2‐AFC test on a cohort of five participants (Exp E) (Figure S2e, Supporting Information). Participants' accuracy was significantly higher than chance with the MDL (χ^2^(1) = 36.97, *p* < 0.001; *d*′ = 2.62, *d*′ 95% CI [1.45,3.79]; Table S3, Supporting Information), but not with the red laser pointer (Figure 3c), probably due to its lower power. In the MDL condition, all but one participant was at or above 75%. For the chopped MDL source, we also added sham trials in which the non‐inked finger was stimulated to verify that participants could only determine which hand was stimulated when stimulation was on the inked finger. Without ink, participant accuracy was no different than chance (*p* = 0.65; Table S4, Supporting Information), suggesting that chopped light from the MDL laser could not be felt without black ink (Figure S7, Supporting Information).

#### 2.2.2 Modulated Light Source

The MDL source was then tested while being modulated, rather than mechanically chopped (Figure 4a). To modulate the light, we input a 20 Hz, 2 ms pulse width signal from a function generator with the MDL output in modulation mode, rather than continuous wave (CW) (Figure 4b). We performed a 2‐AFC experiment on a new cohort of 12 participants.

We added sham trials in which the non‐inked finger was stimulated to verify that participants could only determine which hand was stimulated when stimulation was on the inked finger. We first asked whether participants reported experiencing stimulation only when the inked finger was stimulated (i.e., detection accuracy). Participants' detection accuracy was significantly higher than chance (χ^2^(1) = 302.26, *p* < 0.001; *d*′ = 2.11, *d*′ 95% CI [1.84,2.38]) (Figure 4c). We additionally characterized individual participant performance and found that 8/12 participants performed significantly better than chance (all *p*_*adj*_ < 0.05), two participants performed significantly better than chance only when a multiple comparisons correction was not applied (*p* = 0.006 and *p* = 0.03), and two participants were not significantly better than chance (*p* = 0.19 and *p* = 0.47). The 8/12 participants whose performance was significantly better than chance also performed better than the 75% accuracy criterion. We next asked whether participants could report which hand was stimulated (i.e., discrimination accuracy) when the non‐inked hand was stimulated; consistent with our expectations, participants' discrimination performance (left vs right hand) was not significantly different from chance (χ^2^ (1) = 0.01, *p* = 0.92; *d*′ = 0.01, *d*′ 95% CI [–0.29,0.32]) (Figure 4d). In contrast, when the inked hand was stimulated, (Exp F) (Figure S2f, Supporting Information), participants' discrimination accuracy was significantly better than chance (χ^2^ (1) = 231.78, *p* < 0.001; *d*′ = 2.78, *d*′ 95% CI [2.34,3.21]) (Figure 4c). We additionally characterized individual participant performance and found that 11/12 participants performed significantly better than chance (all *p*_*adj*_ < 0.05), whereas one participant was not significantly better than chance (*p* = 0.84). Consistent with the detection data, the participant whose discrimination accuracy (left vs right) was not significantly better than chance was one of the two participants whose detection accuracy (present vs absent) was not significantly better than chance. Interestingly one participant was not significantly more accurate than chance on the detection task (60.4% accuracy, *p* = 0.19) but was significantly more accurate than chance on the discrimination task (83.3% accuracy, *p* = 0.002), suggesting that there were trials in which the participant reported not feeling stimulation, but could accurately guess the location of stimulation.

Lastly, we tested for sensation quality on the inked distal and proximal phalanges of the participant's dominant hand (Exp D) (Figure S2d, Supporting Information). There was a significant difference in the sensations reported at each stimulation location, χ^2^(5) = 12.5, *p* = 0.03. Across all participants, the most commonly reported primary sensation at the distal phalanx was vibration (55.2%), followed by pressure (17.7%), and heat (14.6%). The most commonly reported primary sensation at the proximal phalanx was still vibration (41.7%), but with heat close behind it (33.3%). Post‐hoc analysis of the Bonferroni‐corrected χ^2^ residuals^[^ ^45^ ^]^ suggests that at the distal phalanx (vs proximal phalanx) there were significantly fewer reports of heat (Z = –3.04, *p*_*adj*_ = 0.01). The most commonly reported secondary sensation at the distal phalanx was heat (44.79%) with pressure far behind (19.79%). Interestingly, the proximal phalanx had a near three‐way tie between pressure (26.04%), vibration (25%), and itch (21.88%).

### 2.3 Incoherent Light Experiments

Given the brightness, availability, and ease of programming the light from a conventional light projector, we sought to investigate whether photoacoustic mechanosensation could be felt using incoherent light. To test this hypothesis, we used a commercial digital light processing (DLP) projector (i.e., a tabletop slide projector) with a Fresnel converging lens 17.8 cm away from the projector output lens (Figure 4e). The focal point was approximately 18 cm away from the Fresnel lens. With the projector oriented such that the beam was directed downward, the subject's finger was fixed at the focal distance. Black ink was used as an absorber on the skin and green pulsed light (15 Hz, 25% duty cycle) (Figure 4f) was played. Participants' detection accuracy was significantly higher than chance (Wald Z = 16.1, *p* < 0.001, *d*′ = 2.38, *d*′ 95% CI [2.08,2.67]) (Figure 4g). Overall, 11/12 participants performed significantly better than chance and the 75% criterion in detection accuracy. Similar to the modulated MDL experiment, we added sham trials in which the non‐inked finger was stimulated, to verify that participants could only determine which hand was stimulated when stimulation was on the inked finger. As expected for those trials, participant performance was not significantly different from chance (χ^2^(1) < 0.01, *p* > 0.99; *d*′ = 0.00, *d*′ 95% CI [–0.30,0.30]) (Figure 4h).

Next, we measured 2‐AFC performance on the subset of data in which the inked finger was stimulated (Figure S2f, Supporting Information). Participants performed significantly better than chance (Likelihood Ratio Test, χ^2^(1) = 252.01, *p* < 0.001; *d*′ = 2.99, *d*′ 95% CI [2.52,3.46]) (Figure 4h). We looked at individual participant performance and found that 11/12 participants performed significantly better than chance (all *p*_*adj*_ < 0.005), whereas one participant was not significantly better than chance (*p* = 0.54).

Finally, we tested for sensation quality on the inked distal and proximal phalanges of the participant's dominant hand (Exp D) (Figure S2d, Supporting Information). The primary sensation felt at the distal phalanx was selected as vibration 57% of the time across participants, followed by heat (26.0%). At the proximal phalanx, 44.8% of the responses for the primary sensation felt were vibration, while heat was selected 28.1% of the time. Like the modulated MDL and OPO, the highest proportion of reported secondary sensations was heat at the distal phalanx (33.33%). At the proximal phalanx, the projector source elicited mostly vibration (28.13%), pressure (25%), and heat (23.96%).

## 3 Discussion

The psychophysical results in this study have demonstrated a robust tactile sensation using all of the light sources, except for one, with the use of a dye as an optical absorber. The sources ranged from a high‐cost and high‐power photoacoustic imaging system to a low‐cost commercially available DLP projector. The OPO laser experiments demonstrated a variety of capabilities amenable to using haptic photoacoustics. Namely, we showed consistent detection of the stimulus on inked fingers, with 9 of the 10 participants scoring 100% accuracy. Participants were able to discern direction of travel of the beam across their skin, showing promising implications of using haptic photoacoustics as a means of encoding information, such as numbers, shapes, or letters. Also unique to the experiments with the OPO, we show the versatility of the light wavelength (680 nm, 808 nm) and optical absorber type (black ink, ICG), by showing that participants detect the stimulus more often than predicted by chance, without any significant influence from particular wavelengths or absorber types. This finding is also notable in that ICG and 808 nm light are both much less visible than the black ink and 680 nm light, which means that such photoacoustic haptic effects can be produced discreetly if need be.

When comparing the chopped MDL and red laser pointer, we found that there is some power threshold that determines whether or not the chopped light can be perceivable as a tactile sensation. We know this because the chopping parameters were identical for each of these light sources, yet participants could perform significantly higher than chance in a 2‐AFC test, while the same participants could not with the red laser pointer. Given the identical chopping parameters, this observation leads us to hypothesize that the difference in irradiance is what is responsible for discrepancies in detection of the stimuli.

We note some idiosyncrasies of the effects produced using different methods of pulsing the light. Anecdotal reports from the participants suggest that sensations from the chopped MDL were more likely to be perceived as heat than with the OPO system, possibly owing to a significantly longer duty cycle (i.e., dwell time) due to the homemade mechanical chopper: ca. 12.5% versus 0.00002%. In instances in which we chopped the light mechanically, the duty cycle has a dependency on the relative size of the optical window (e.g., between fan blades), which can cause some physical constraints in the design. Additionally for the same optical window, varying the duty cycle would require a change in frequency, introducing inconsistencies. To produce smaller and more consistent pulses, digital modulation was used for the next experiment.

**Figure 5:**
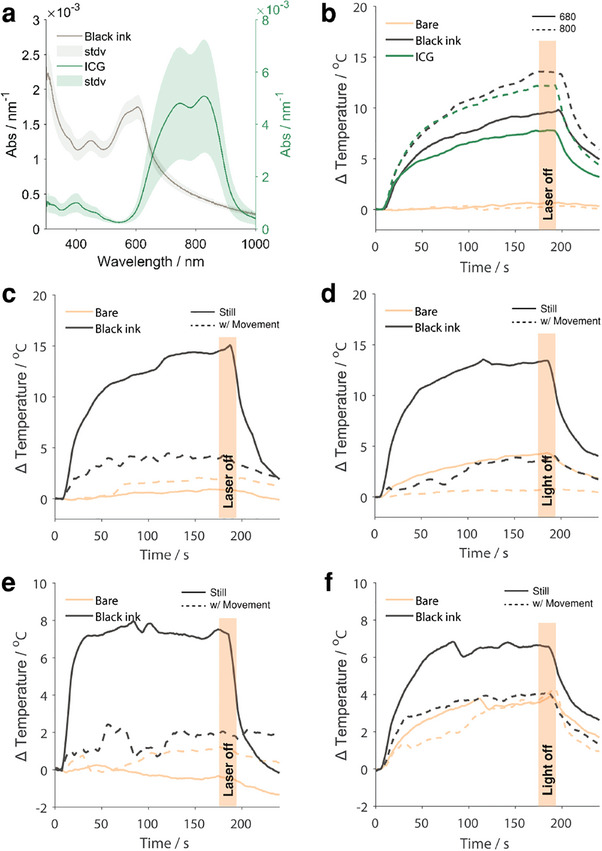
Characterization of dyes' light absorption. a) Absorbance spectra for ICG and black ink. b–f) Thermographically measured temperature versus time with and without dyes with light stimulation: b) on the fingertip using OPO system c) on porcine skin samples using MDL source d) on porcine skin samples using DLP source e) on the fingertip using the MDL source f) on the fingertip using the DLP source. MDL and DLP sources include stationary and moving light temperature profiles.

While the DLP had two more participants that performed above the 75% accuracy criterion compared to the modulated MDL, neither detection nor discrimination accuracy were significantly different between the two sources (Detection: χ^2^(2) = 3.46, *p* = 0.18; Discrimination: χ^2^(2) = 0.44, *p* = 0.80). This is particularly interesting given the properties of the sources. The DLP produced an incoherent spectrum of light modulated into two peaks per period Figure 4f. Given such parameters, we expected the DLP projector to be less effective in delivering perceivable tactile stimulation. However, the absorbance of the DLP light could have been enhanced by the intrinsic absorbance of the skin in the visible region. This observation could be seen as an increase in the temperature of DLP‐illuminated bare skin (anecdotally shown in **Figure** **5**d). The participant who was not significantly more accurate than chance at reporting left versus right in the DLP projector experiments was the participant whose detection performance was not significantly better than chance—and was also the participant with at‐chance performance with the MDL laser. The high detection accuracy experiments for both sources further support the necessity to place dye on the skin to increase the absorption and subsequent tactile sensation. With the demonstrated efficacy of both black ink and ICG (Figure 5a) in the OPO experiments, the options for optical absorbers and corresponding light wavelengths can potentially be vast.

To qualitatively evaluate what types of sensations were being detected, we looked at sensation quality across three of the main light source types in this study: OPO, modulated MDL, and the DLP projector. Across all three sources, we consistently saw more reports of vibration as the primary sensation at the distal phalanx (55–58%) versus the proximal phalanx (41–44%). Heat was the most commonly reported secondary sensation for both distal and proximal phalanges for all three sources, with the exception of the proximal phalanx for the MDL and DLP projector; in this case, pressure and vibration were reported as secondary sensations more often than heat. For the OPO laser, heat was the most commonly reported secondary sensation at both locations compared to the other sensations. This is likely due to the higher power of the OPO laser, and the fact that the beam was not moved around during stimulation as quickly as the modulated MDL and DLP projector were, since the larger rectangular beam of the OPO covered most of the finger during stimulation. The acoustic waves generated are mediated by transient absorption and photothermal conversion of the optical energy, and thus the possibility remained that the effect could be perceived as thermal. This difference in perception may be explained by the reduced concentration of mechanosensory neurons—particularly the Meissner's corpuscles sensitive to frequencies in the range of the 20 Hz repetition rate of the laser—closer to the palm.^[^ ^46^ ^]^

Consistent with OPO and MDL stimulation, vibration was reported more often across all participants for the DLP projector. However, unlike with the OPO and MDL stimulation, there was no significant difference in the sensations reported at each stimulation location, χ^2^(5) = 4.00, *p* = 0.55. The absence of a statistically significant difference in sensations between the distal and the proximal phalanges here might be due to the light source and pulsing parameters of the DLP system. As shown in Figure 4f, the DLP projector produces a double peak pulse train which is due to the superimposition of the DLP system's frame rate and the digital commands given by the computer. Additionally, the non‐monochromatic nature of the green DLP light (see Figure S5, Supporting Information) and its interaction with the black ink may have produced inconsistent or indiscernible sensations among participants. Further investigation is required to better understand the discrepancies observed in sensation quality between the DLP projector and the coherent light sources.

## 4 Conclusion

The in situ conversion of optical stimulus into a mechanical sensation, while based on a century‐old effect, represents a potentially powerful modality of neural activation in humans. The effect explored here is robust and highly tolerant of a range of physical parameters such as wavelength, pulse rate, pulse width, and dye sensitizer. It was shown that a thin layer of optical absorber on the skin enhances the mechanical sensation. A key advantage of a system based on in situ generation of mechanical forces in the skin includes the ability to project the stimuli without physical contact. Moreover, the use of optical signals opens the door to the simultaneous stimulation of multiple types of afferent fibers. For example, by using overlaid variations in frequency and pulse width, it may be possible to activate multiple types of mechanically and thermally responsive neurons. Using existing DLP projector technology, where changes in light intensity and pulsing can easily be digitally controlled, makes generating this effect significantly more accessible and amenable to modifications. The DLP system also demonstrates the additional low‐cost and optical safety advantages of using cheap, incoherent light sources in photoacoustic mechanosensation.

Advancing such abilities can facilitate the development of “tactile holography” which could have applications in medical training, physical therapy, pain management, complementary healthcare, remote operation, consumer electronics, and various applications of virtual and augmented reality. There is still, however, much to be learned about the spatial discrimination of this effect, using various dye patterns and light projections. Moreover, such techniques are potentially applicable to mechanobiology in applying high‐precision forces to cells and tissues in vitro, as current micro poking tools require high precision manipulation to make physical contact and exert forces on cells.^[^ ^47^, ^48^ ^]^ In particular, in the neurobiology and neuropsychology of touch and interoception, the ability to activate mechanically responsive cells and tissues without genetic engineering (i.e., optogenetics) or physical contact may open the door to new kinds of assays both in vitro and in vivo.

Previous studies have attempted to model or simulate similar tactile phenomena as well as characterize it through direct measurements. Nevertheless, there still is no comprehensive understanding of the mechanism of mechanotransduction for the effect, and there remains ambiguity on the receptors responsible. Developing a deeper understanding of the mechanics and establishing a robust biophysical model would be one of the first steps in expanding the applicability of this effect. On that basis, the stimulation parameters could be optimized for the various technological applications to which it may be suited.

## 5 Experimental Section

### Psychophysical Design

All participants gave informed written consent and this study was approved by the Institutional Review Board of UC San Diego (#804228). The first set of experiments (Experiments A‐D) were conducted using a commercial nanosecond optical parametric oscillator (OPO) laser for photoacoustic imaging (Vevo F2 LAZR‐X). The applied stimulus had a pulse width of 10 ns with a pulse frequency of 20 Hz, which was the only pulse repetition rate offered by the device. All four experiments used a wavelength of 680 nm, except for Experiment B, in which 800 nm was also used.

Experiments E and F were conducted using the chopped MDL source using the handheld chopper (Figure S6, Supporting Information). The 6.25 ms pulses of light were at a frequency of 20 Hz. There were five participants for experiments E and F using the handheld chopper. The modulated MDL source was used on 12 additional participants for experiments D and F. The pulses of light were still at 20 Hz, but with a pulse width of 2 ms. The DLP projector was also used on the same 12 participants as the modulated MDL experiments. Experiments D and F were repeated using the DLP projector, with a pulse width of 16.7 ms and a frequency of 15 Hz. The pulse repetition rate was limited to 15 Hz due to hardware limitations of the projector. The light that was pulsed from the projector was the standard color green.

The psychophysical designs for all of the experiments conducted in this study are shown in Figure S2 (Supporting Information). All participants were blindfolded and wearing foam earplugs with noise‐cancelling headphones. For each experiment, participants were given the question to be asked prior to the start of the experiment and were prompted to answer the question with a tap on the shoulder at the end of each trial.

1 **Experiment A**: The initial experiment design (Figure S2a, Supporting Information) is a 2‐alternative forced‐choice test aimed at detecting the stimulus using black ink applied to both index fingers. During each trial, the optical stimulus was presented on one of the index fingers. Participants completed a total of 30 trials. On each trial, they were asked, “On which finger did you feel a sensation?” The only acceptable answers were “left index” or “right index.” The trial order was randomized for each participant.
2 **Experiment B**: In this experiment, a four alternative forced choice paradigm was employed to examine the identification of motion direction elicited by the applied stimulus. The dominant hand's distal and middle phalanges were marked with black ink. To assess the participant's perception, the investigator swept the stimulus in four distinct directions (Figure S2b, Supporting Information): front to back, back to front, left to right, and right to left.
  Each participant underwent ten iterations of the four possible directions, presented in a random order using a block design (n = 40 trials per participant). After the stimulus application in each trial, participants were asked, “In which direction did you feel the stimulus sweep?” This procedure allowed to explore the motion perception patterns effectively.
3 **Experiment C**: An experiment was conducted to investigate the impact of different ink types and wavelengths on stimulus detectability (Figure S2c, Supporting Information). Two inks were tested: black ink and indocyanine green (ICG) dye suspended in ethanol (4 mg mL^−1^). The experiment also explored two wavelengths: 680 and 800 nm.
  For each participant, ICG ink was applied to the distal phalanx of their middle finger, while black ink was applied to the distal phalanx of their index finger, both on their dominant hand. With the two wavelengths, four stimulus conditions were established, representing all possible ink/wavelength combinations: ICG with 680 nm, ICG with 800 nm, black ink with 680 nm, and black ink with 800 nm.
  Each participant experienced seven iterations of the four stimulus conditions in a block design, which were randomly ordered, resulting in a total of 28 trials per participant. After each stimulus application, the participants were asked, “On which finger did you feel the sensation?” Acceptable answers were “middle finger” or “index finger.”
4 **Experiment D**: An experiment was conducted to discern the types of sensations experienced by the participants (Figure S2d, Supporting Information). To achieve this, each participant's dominant hand's distal and proximal phalanges was marked with black ink. The stimulus was then randomly applied to either the distal or proximal phalanx in a total of 30 trials. During each trial, the participants were asked “What was the primary sensation you felt?” followed by “What was the secondary sensation you felt?” after the stimulus application. They had six possible sensations to choose from: heat, cool, pressure, vibration, itch, and pain.
(5) **Experiment E**: To determine whether participants could detect stimulation with different lasers (MDL 808 nm 2 W and red pointer 650 nm), an experiment was conducted to apply two different laser stimuli on the participants' inked fingers (Figure S2e, Supporting Information). The MDL 808 nm laser and red pointer 650 nm laser are referred to as laser 1 and laser 2, respectively. The participant's distal phalanges of their index fingers were marked with black ink. There were four possible stimulus conditions: left index with laser 1, right index with laser 1, left index with laser 2, and right index with laser 2. The four conditions were counterbalanced in a total of 20 trials.
6 **Experiment F**: An experiment was conducted to determine whether participants could detect stimulation without ink. The MDL 808 nm laser was used with the handheld chopper, modulated MDL, and DLP projector for this experiment. The participant's middle and index fingers on both of their hands were used for the experiment (Figure S2f, Supporting Information). Each hand had one of the two fingers marked with black ink (distal phalanx) and the other one bare. The four possible ink placements were counterbalanced across participants, with one participant condition repeated since we had five total participants. The stimulus was applied to each of the four fingers in random order for a total of eight blocks (n = 32 total trials). After applying the stimulus, the participant was instructed to answer two questions: 1) “Did you feel a sensation?” and 2) “On which hand was the sensation felt?” Even if the answer was “no” to the first question, an answer had to be given for the second question.

### Experimental Setup—OPO Experiments (Experiments A–D)

A chair and bench top were prepared for the human subject, fitted with a platform to place their hands on. The optical parametric oscillator laser of a VisualSonics Vevo F2 LAZR‐X was connected to the mediumsize fiber optic cable. The two ends of the cable were taped together for easier handling. It delivered 10 ns pulses of 680 or 800 nm wavelength at a repetition rate of 20 Hz (duty cycle = 0.00002%). The diverging beam was spread to illuminate a rectangular area of 2.7 cm^2^ when held 2 cm from a surface. Participants were seated and blindfolded. Audible frequencies emanating from the skin (Figure S1, Supporting Information) during the experiments were blocked with foam earplugs combined with noise‐canceling headphones.

The subjects were asked to place in foam earplugs and a blindfold. The subjects' hands were cleaned from any residues and the dye was applied corresponding to each experiment. Two coats of ICG dye were applied using a cotton swab and allowed to dry between coats. The black ink was directly applied onto the subjects' finger/s using a permanent marker (Sharpie Fine Point—Black). Noise‐canceling headphones (RUNOLIM WH301A) were placed on the subjects' ears.

A Python script was used to randomize the stimulation parameters.

### Experimental Setup—MDL Experiments

The participant preparation for the MDL experiments was identical to that of the OPO experiments.

1) Mechanically chopped light (Experiments E,F): The mechanical laser chopper setup was built using a simple DC motor controlled using an Arduino microcontroller fitted with a motor control shield. Parts were designed in a 3D CAD software and printed on an FDM 3D printer using polylactic acid (PLA) and flexible thermoplastic polyurethane (TPU) filaments. The laser socket was made to fit the various laser sizes examined allowing for easy swapping during the experiment. For thin fiber optic cables custom adapters were made to ensure a tight fit and minimize deviation.

2) Modulated light (Experiments D,F): The laser modulation signal was from a functional waveform generator (SIGILENT SDG 2042X) connected to the input of the MDL power supply. The MDL power supply was set to modulation mode, rather than CW. The fibre optic cable coming out of the MDL laser was fixed using a laboratory stand with a clamp attachment, so that the output of the fibre optic was pointed vertically downward. The participant's hand was placed so that the palm was faced upward on top of a piece of foam for comfort. The height of the fibre optic cable output was positioned so that it was 1.5 cm above the surface of the participant's fingers.

### Experimental Setup—DLP Projector Experiments (Experiments D,F)

The participant preparation for the DLP projector experiments was identical to that of the OPO and MDL experiments.

The DLP projector (OPTOMA HD146X) was fixed to a lab jack with 3D‐printed parts to hold the projector to shine vertically downward. The projector was strapped to the stand using large velcro straps. The fresnell lens was fixed at a distance of 17.8 cm from the output of the projector using 3D‐printed parts. The lens captured less than half of the projected area at the used distance. A standard green projection was played as a video that was generated using Python OpenCV library. The color was chosen to reduce the time average intensity of the output; white (combination of all colors) light being the most intense. The height of the projector, determined by the height of the lab jack, was set so that the focal point of the lens landed on the surface of the participant's fingers. The participant's hand was placed so that the palm was faced upward on top of a piece of foam for comfort.

### Characterization—Dye Absorbance

ICG solid was dissolved into ethanol (4 mg mL^−1^), and filtered through a 0.45 µm, 13 mm filter to remove aggregates. For a controlled comparison, black ink was extracted from a permanent marker (Sharpie Fine Point—Black) by placing the tip of a fresh marker into a vial of ethanol (20 mL) overnight. Glass slides (1″ x 1″) were cleaned in a sonication bath using the following wash sequence (Alconox solution, water, isopropanol). The slides were then dried and treated in a plasma cleaner (400 mTorr air, 3 min, 30 W) to activate the surface. The solution was spun on the glass slides at 600 rpm accelerated at 300 rpm s^−1^. Three samples were prepared of each dye.

The absorption spectra of each sample were obtained between 300 and 1000 nm (Aligent Cary UV–Vis Spectrophotometer). The thickness of the samples was evaluated at two points using ellipsometery (J.A. Woollam M‐2000D Spectroscopic Ellipsometer). The thickness was used to normalize the absorption spectra. The absorption spectrum of a permanent marker drawn on a glass slide was also obtained to rule out any inadvertent changes to the marker ink during extraction.

### Characterization—Audio Measurements

A willing subject's index, middle, and ring finger were prepared in a manner similar to the thermography experiments. The hand was placed in a box padded with noise insulation. A lapel microphone without a pop filter was placed inside the box behind the location of the laser to avoid any photoacoustic effects on the microphone surface. The audio jack of the microphone was connected to a SONY Alpha 7R camera. The laser was applied on the different dyes and bare skin while the audio was being recorded. This experiment was repeated for bare and inked (ICG and black ink) fingers for both wavelengths. To capture the ambient noise generated by the laser, the laser probe was pointed away from the subject and held far away (>1 m) from any surfaces. The audio was collected from the video recordings. A 10kHz high pass filter (24 dB) was applied to the audio data to remove the background machine noise.

### Characterization—Thermography

A willing subject's index and ring fingers were coated with black ink and ICG respectively, the middle finger was set as the control (bare). A thermographic camera (HIKMICRO Pocket 2) was affixed on a stand to measure the temperature of the finger tip at the laser's area of incidence. The ambient temperature was input into the camera settings and the emissivity was validated by comparing the temperature with a thermocouple output (Fluke t3000 FC). Each finger was illuminated with the laser source using the OPO with identical wavelengths and pulse settings used in the psychophysical experiments for ca. 3 min. The laser was then turned off to observe the cooling effect. The thermographic data were extracted from the video footage using MATLAB Image Processing Toolbox and Computer Vision Toolbox. The data was smoothed using a moving mean (20 samples) and plotted against the time signature of the frame.

### Characterization—OPO Power Measurements

To first measure pulse frequency, an oscilliscope (TDS2022C) was connected to a biased photodetector (Thorlabs DET10A2 200 ‐ 1100 nm). The OPO laser was pointed directly at the center of the detector sensor and was set to pulse 680 nm wavelength light. The oscilloscope captured the sensor power readings (Figure S8, Supporting Information). Confirmed by the manufacturer, the OPO laser, while at a 20 Hz pulse repetition rate, skips a pulse every 4 pulses, resulting in 16 pulses every second. To calculate a peak fluence, a beam spot at 1.5 cm away from the target of 2.7 cm^2^ was estimated, and calculated a peak fluence of 13.3 mJ cm^−2^ given the technical specification of 45 mJ peak energy for a 20 Hz 4–6 ns pulse, provided by the OPO manufacturer.

### Characterization—MDL Power and Pulse Measurements

The output power of the MDL laser, immediately after exiting the optical laser, was calculated to be ≈2.29 W. This calculation was based on a maximum source power of 2.5W, with an estimated power loss of 9.95 % through the optical fiber. To observe the pulse profiles for the chopped and modulated MDL light, a digital power meter (Thorlabs PM100D) and a compatible photodiode sensor (Thorlabs S120VC) were fixed on an optical platform, in the direct line of sight of the optic fiber cable output. The modulation or chopping were started, and laser energized. The output signal was observed and collected using a digital oscilloscope (RIGOL DS1054 Z) and then normalized by the maximum power. Modulation was varied from the parameters used for stimulation to ensure the chosen modulation frequency did not affect maximum power.

The beam diameter was estimated at $\frac{1}{e^{2}}$ of the maximum intensity. The beam was approximated to have a near‐gaussian distribution with speckle observed. To obtain the $\frac{1}{e^{2}}$ beam diameter, the fiber optic beam was placed 1.5 cm away from the photodiode, and the current setting was lowered to avoid sensor damage. An optical chopper wheel was used to scan the beam (Thorlabs MC2000B) at 20Hz. The blade (Thorlabs MC1F10HP) was placed in between the beam and sensor and an intensity profile was obtained. The intensity profile was then used to calculate the diameter (1.28 cm). The fluence was calculated using the pulse parameters and beam diameter.

### Characterization—DLP Projector Power and Pulse Measurements

All measurement components were secured to an aluminum optical breadboard with the projector‐to‐lens distance and lens‐to‐photodiode distances set to 17.8 cm. To avoid input saturation, a neutral density filter (NE60A) with an optical density of 6.0 was attached to the diode input. The power meter's attenuation was adjusted to 60.00 dB to correct for the attenuation from the ND filter. Given that the photodiode exhibits a linear responsivity curve within the visible light regime, the measurement was set to be at a wavelength of 540 nm on the power meter, corresponding to the central wavelength of the UV–vis absorption spectrum of the projector's green color filter (Figure S5, Supporting Information). During data acquisition, the projector was set to a standard green screen, and the average power reading was recorded. A “continuous” green screen showed a pulsing pattern consistent with the DLP refresh rate which was averaged by the power meter. For digital pulse characterization, the projector and photodiode were positioned 35.6 cm apart, with no other optical components interposed. The pulse waveforms were captured on an oscilloscope connected to the output of the power meter for both “continuous” and pulsed light (operating at 15 Hz with a pulse width of 16.7 ms). The photodiode power sensor, digital power meter, and oscilloscope used for these measurements were identical to those mentioned previously. Using the power measured for the “continuous” DLP projector light (310 mW), we calculated a peak fluence of 10.3 mJ cm^−2^ using the 15 Hz pulse repetition rate, 16.7 ms pulse width, and measured beam spot (≈0.5 cm^2^).

### Statistical Analysis

1. **Experiment A**: To characterize overall participant performance, a logistic mixed effects regression was fitted with dependent variable of participant response (left or right), fixed effect of stimulation location (left or right), and random effect of participant. Participant accuracy was assessed using a Likelihood Ratio Test to compare the full model to a nested model in which the stimulation location term was removed. D‐prime estimate and confidence interval were derived from the independent variable coefficient using the method described in ref. [49]. To characterize individual participant performance, we used a separate binomial test for each participant, with a 95% confidence interval determined via the Clopper–Pearson method.^[^ ^50^ ^]^ A Bonferroni correction was applied for multiple comparisons.
2. **Experiment B**: To characterize overall participant performance, a logistic random effects regression was fitted with dependent variable of trial outcome (correct or incorrect), and random effect of participant. Participant accuracy was assessed using a Likelihood Ratio Test to compare the full model to a nested model in which the intercept term was removed (fixed to chance performance). D‐prime estimates and confidence intervals were derived from the model estimate of accuracy using the table in ref. [51]. To characterize individual participant performance, we used a separate binomial test for each participant, with 95% confidence intervals determined via the Clopper–Pearson method. A Bonferroni correction was applied for multiple comparisons.
3. **Experiment C**: To characterize the effect of ink type and wavelength on participant performance, a logistic mixed‐effects regression model was fitted with dependent variable of trial outcome (correct or incorrect), fixed effects of ink type (Black ink vs ICG), wavelength (680 vs 800 nm), and their interaction, and a random effect for participant. The effect of choice of ink and wavelength was assessed using a Wald Z test of each model coefficient (Table S1, Supporting Information). Participant accuracy was assessed using Z tests of the estimated marginal means of accuracy for each combination of ink and wavelength.
4. **Experiment D**: Response proportions were computed separately for primary and secondary sensations within each sensation type. Subsequently, the proportions of primary and secondary responses were combined and overall response proportions were calculated for each sensation type encompassing both primary and secondary sensations. To determine whether the reported sensations significantly differed by stimulation location, three 2 (proximal/distal) x 6 (vibration/heat/pain/cold/itch/pressure) χ^2^ tests of independence were run. To characterize the contribution of each sensation to the omnibus χ^2^ statistic. Post‐hoc, Bonferroni‐corrected Z tests of the χ^2^ residuals was conducted using the method described in ref. [45].
5. **Experiment E**: For the chopped MDL and red laser pointer (Figure 3), a logistic mixed effects regression was fitted on sham trials only with dependent variable of trial outcome (correct/incorrect), fixed effect of laser (MDL 808 nm vs red pointer 650 nm), and random effect of participant. The effect of choice of laser was assessed using a Wald Z test of the model coefficient. Participant accuracy was assessed using Z tests of the estimated marginal means of accuracy for each laser.
6. **Experiment F**: For the chopped MDL and red laser pointer (Figure S6, Supporting Information), a logistic mixed effects regression was fitted with dependent variable of trial outcome (correct/incorrect), fixed effect of ink (present/absent), and random effect of participant. The effect of choice of laser was assessed using a Wald Z test of the model coefficient. Participant accuracy was assessed using Z tests of the estimated marginal means of accuracy for each laser.
  For the modulated MDL and DLP projector (Figure 4), to test whether participants could detect presence or absence of stimulation, a logistic mixed effects regression was fitted on all trials, with dependent variable of participant response (stimulation present vs absent), fixed effect of stimulation (inked vs non‐inked; i.e., stimulation vs no stimulation), and random effect of participant. To verify that participants could not perceive stimulation of the non‐inked finger (i.e., that it was truly analagous to “no stimulation”), a logistic mixed effects regression was fitted on the sham (non‐inked) trials only, with dependent variable of participant response (left or right), fixed effect of stimulation location (left or right), and random effect of participant. To test whether participants could identify which finger was stimulated on the stimulation (inked) trials, a logistic mixed effects regression was fitted on the stimulation (inked) trials only, with dependent variable of participant response (left or right), fixed effect of stimulation location (left or right), and random effect of participant. Participant accuracy was assessed using a Likelihood Ratio Test to compare the full model to a nested model in which the stimulation location term was removed. D‐prime estimate and confidence interval were derived from the independent variable coefficient using the method described in ref. [49]. To characterize individual participant performance, a separate binomial test was used for each participant, with 95% confidence intervals determined via the Clopper–Pearson method. A Bonferroni correction was applied for multiple comparisons.

## Conflict of Interest

The authors declare no conflict of interest.

## Supporting information

Supporting Information

## Data Availability

The data that support the findings of this study are available from the corresponding author upon reasonable request.
