# Supplementary material for: Enhanced Dye‐Sensitized Mechanosensation Utilizing Pulsed and Digitally Modulated Light
Source: Adv Sci (Weinh). 2024 Aug 19;11(39):2403690. doi: 10.1002/advs.202403690 (PMC11496992; doi:10.1002/advs.202403690)
Supplement: Supplementary file 1 — Supporting Information [file ADVS-11-2403690-s001.pdf]

ADVANCED SCIENCE
Open Access

Supporting Information

for *Adv. Sci.*, DOI 10.1002/advs.202403690

Enhanced Dye-Sensitized Mechanosensation Utilizing Pulsed and Digitally Modulated Light

*Tarek Rafeedi, Laura L. Becerra, Nicholas Root, Yi Qie, William Brown, Baiyan Qi, Lei Fu, Guillermo Esparza, Lekshmi Sasi, Kabir Kapadia, Romke Rouw, Jesse Jokerst and Darren J. Lipomi**

Supplementary Information

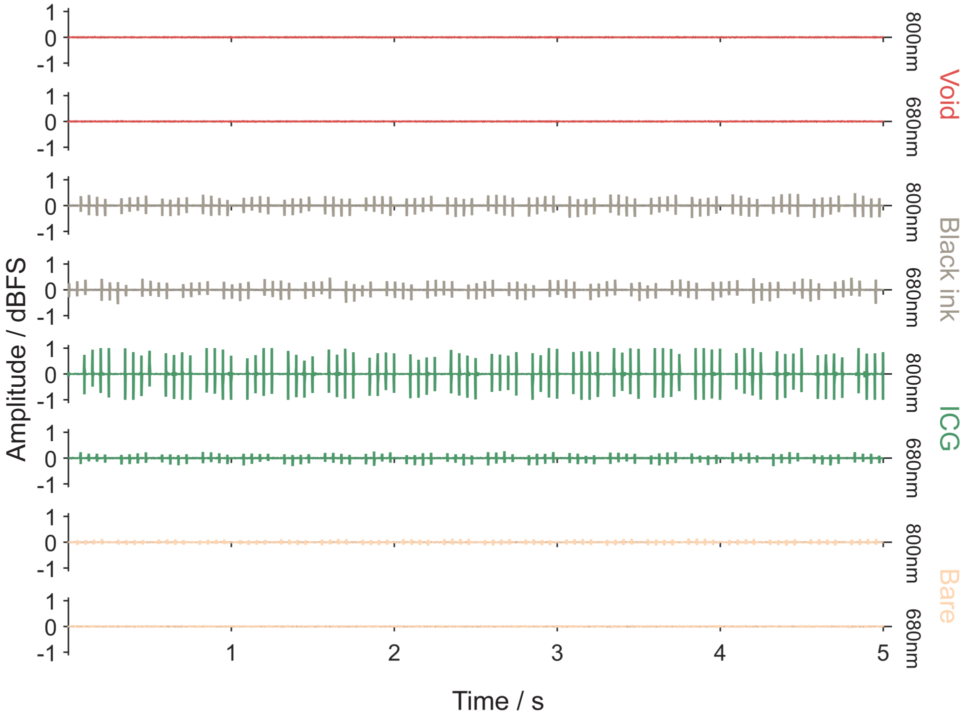

Figure S1: Graphical representation of the audio signals generated by OPO pulses (800 nm and 680 nm) incident on inked (black ink and ICG) fingers, bare finger, and void. Note: The OPO laser skips a pulse every 4 pulses (16 pulses per second), though still pulsing at 20 Hz (see (Figure S 8).

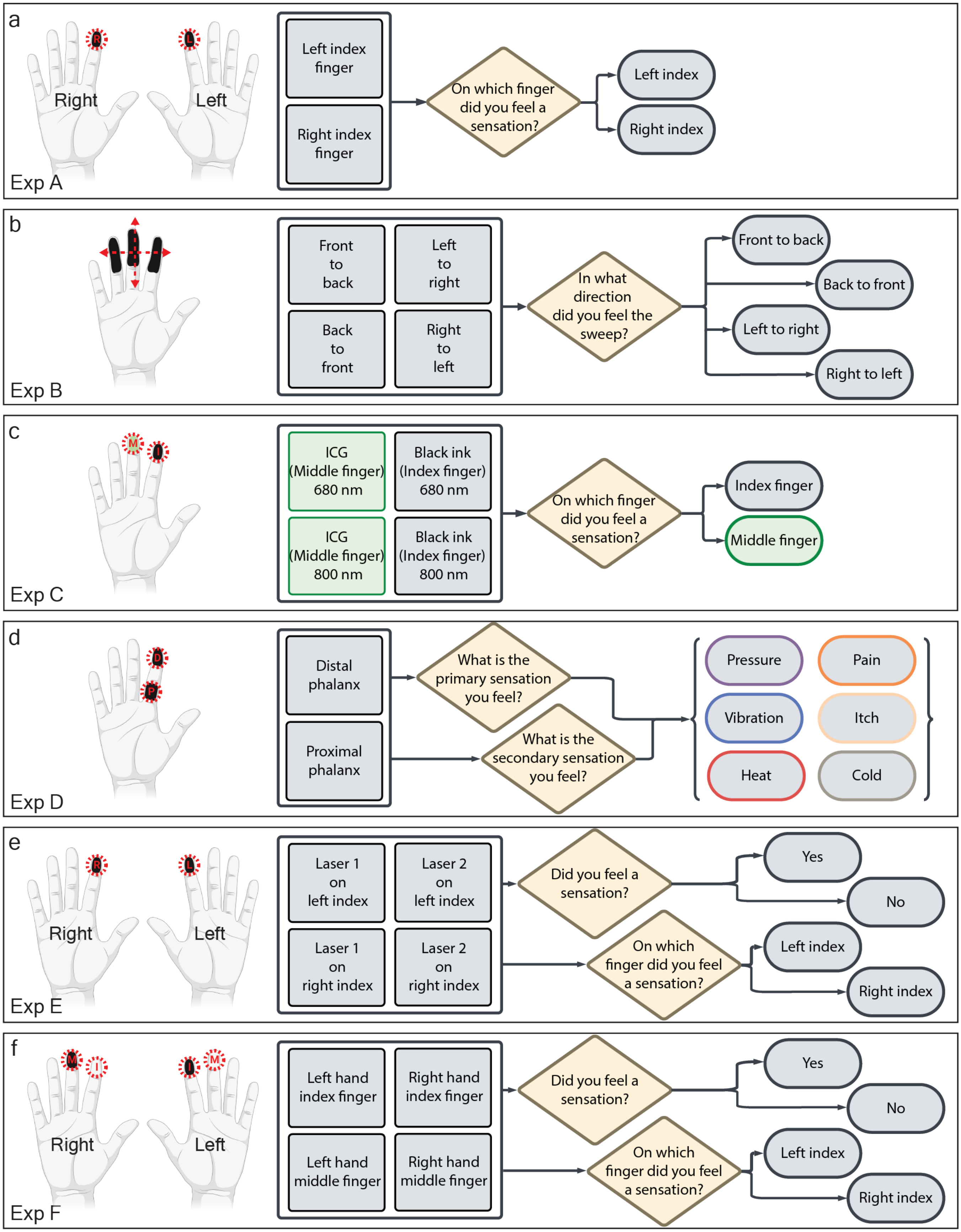

Figure S2: Schematic (left) and flowchart (right) description of laser experiments for: a, Experiment A (OPO) **b,** Experiment B (OPO) **c,** Experiment C (OPO) **d,** Experiment D (OPO, MDL (modulated), DLP) **e,** Experiment E (MDL chopped) **f,** Experiment F (MDL (chopped and modulated), DLP).

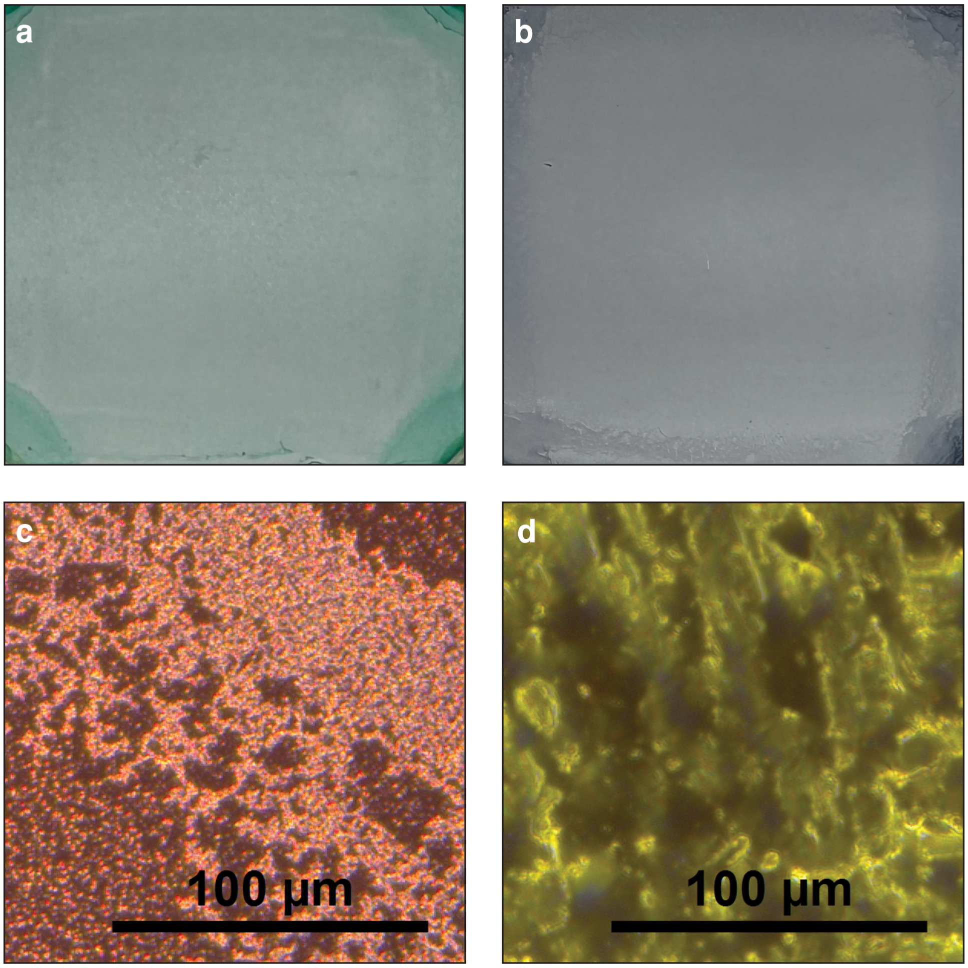

Figure S3: Photographic and optical microscopy images of spin-cast dye films. a, Photograph of ICG film on glass substrate. **b,** Photograph of black ink film on glass substrate. **c,** Corresponding ICG film dark field optical microscopy image. **d,** Corresponding black ink film dark field optical microscopy image.

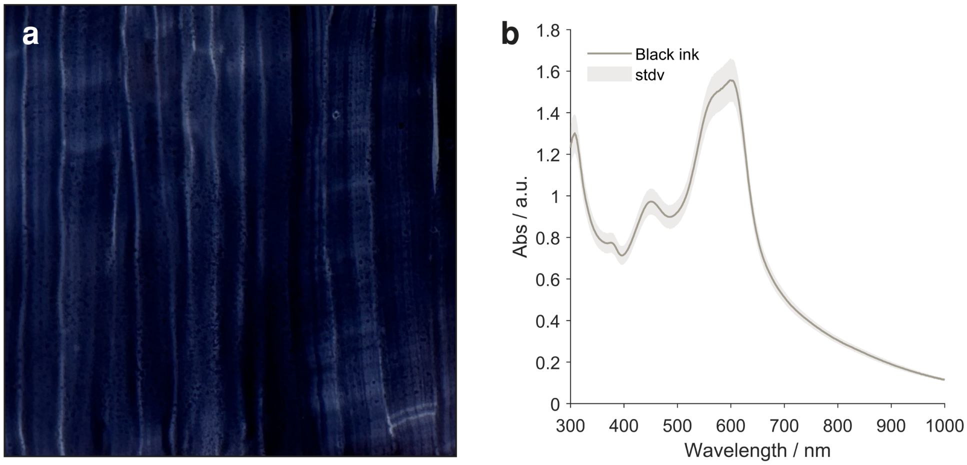

Figure S4: Photographic and absorbance spectrum representations of black ink drawn on a glass slide. a, Photograph of black ink film hand drawn on glass substrate. **b,** Absorbance spectrum of black ink drawn film not normalized by thickness.

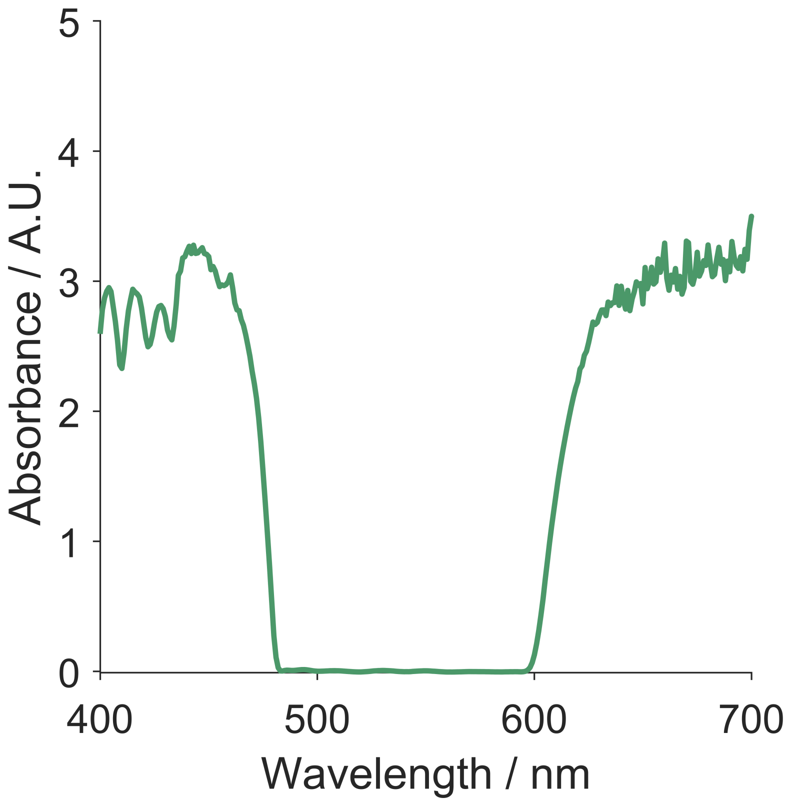

Figure S5: Absorbance spectra of DLP projector green light.

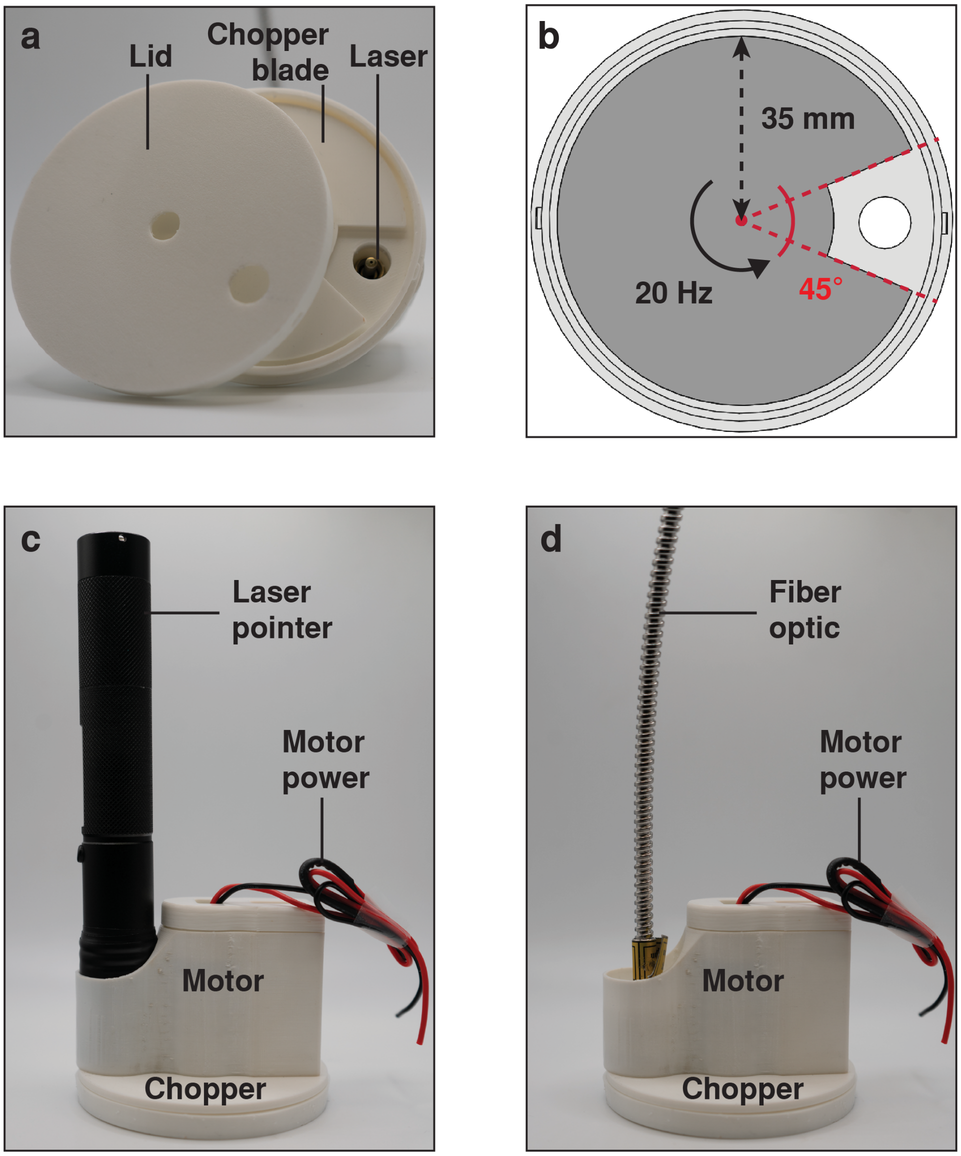

Figure S6: Photographic and schematic details of the hand-held laser chopper. a, Chopper assembly showing the laser aperture, chopper blade, and protective lid. **b,** Chopper blade schematic showing the angle of the chopper window (aligned with aperture) and the diameter of the blade. **c,** Handheld chopper assembled with laser pointer in the socket. **d,** Handheld chopper assembled with fiber optic cable in the socket.

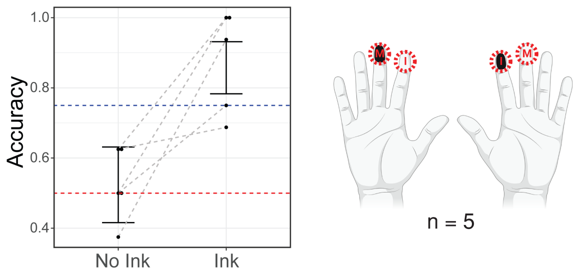

Figure S7: Accuracy of participants' ability to detect the haptic effect with and without ink using chopped MDL light. Error bar confidence intervals and chance performance are the same as those for Fig. 3c

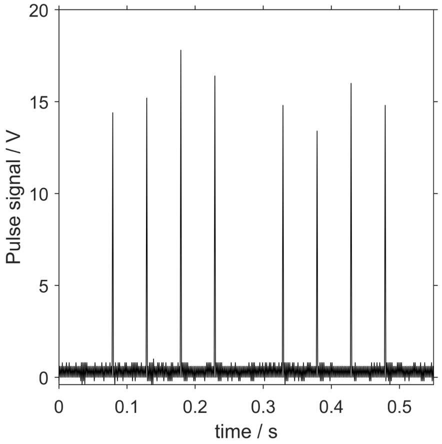

Figure S8: OPO laser power pulse signal.

Table S1: Exp. C: General Linearized Mixed Model (GLMM) results.

Term	Coefficient	Std. Err.	Wald Z	p value
Intercept	7.37	3.22	2.29	0.02
Wavelength (800 nm)	0.47	0.98	0.48	0.63
Ink (Black)	2.52	1.15	2.19	0.03
Ink * Wavelength	0.15	1.49	0.10	0.92

Table S2: Exp. C: GLMM Estimated Marginal Means (EMMs) of each condition. Values are back-transformed from the logit scale. Tests are performed on the logit scale

Wavelength	Ink	Accuracy EMM	Accuracy EMM 95%CI	Wald Z	p value
680	ICG	>0.99	[0.74, 1.00]	2.29	0.02
800	ICG	>0.99	[0.80, 1.00]	2.38	0.02
680	Black ink	>0.99	[0.96, 1.00]	2.86	0.004
800	Black ink	>0.99	[0.98, 1.00]	3.01	0.003

Table S3: Exp. E: GLMM Estimated Marginal Means (EMMs) of each condition. Values are back-transformed from the logit scale. Tests are performed on the logit scale.

Laser	Accuracy EMM	Accuracy EMM 95%CI	Wald Z	p value
MDL 808 nm	0.91	[0.78, 0.97]	4.30	< 0.001
Laser pointer	0.44	[0.28, 0.61]	0.72	0.47

Table S4: Exp. F (Mechanically chopped): GLMM Estimated Marginal Means (EMMs) of each condition. Values are back-transformed from the logit scale. Tests are performed on the logit scale.

Laser	Accuracy EMM	Accuracy EMM 95%CI	Wald Z	p value
No ink	0.53	[0.42, 0.63]	0.45	0.65
Ink	0.88	[0.78, 0.93]	5.76	<0.001

Table S5: Spin-cast dye film thicknesses obtained by ellipsometry. Each film sample was measured at two separate points denoted by the header: "thickness".

	Thickness 1 (nm)	Thickness 2 (nm)	Mean thickness (nm)
		ICG	
Sample 1	76.52	60.09	68.31
Sample 2	65.79	71.03	68.41
Sample 3	48.44	45.07	46.76
		Black ink	
Sample 1	86.05	68.63	77.34
Sample 2	68.26	57.26	62.76
Sample 3	65.37	68.37	66.87

Table S6: Total rectangular area of OPO laser beam projected over different distances.

Distance (mm)	Area (mm^2)
20	ca. 270
100	ca. 990
